# Predicting frailty in older adults using vocal biomarkers: a cross-sectional study

**DOI:** 10.1186/s12877-022-03237-7

**Published:** 2022-07-01

**Authors:** Yu-Chun Lin, Huang-Ting Yan, Chih-Hsueh Lin, Hen-Hong Chang

**Affiliations:** 1 Department of Chinese Medicine, China Medical University Hospital, No. 2, Yude Road, North District, 40447 Taichung, Taiwan; 2 Graduate Institute of Integrated Medicine, China Medical University, No.91, Hsueh-Shih Road, North District, Taichung, 40402 Taiwan; 3 Institute of Political Science, Academia Sinica, 128 Academia Rd., Sec.2, Nankang, Taipei, 115 Taiwan; 4 School of Medicine, College of Medicine, China Medical University, No.91, Hsueh-Shih Road, North District, Taichung, 40402 Taiwan; 5 Department of Family Medicine, China Medical University Hospital, No. 2, Yude Road, North District, Taichung, 40447 Taiwan; 6 Chinese Medicine Research Centre, China Medical University, No.91, Hsueh-Shih RoadNorth District, Taichung, 40402 Taiwan

**Keywords:** Acoustic measures, Frailty, Older adults

## Abstract

**Background:**

Frailty is a common issue in the aging population. Given that frailty syndrome is little discussed in the literature on the aging voice, the current study aims to examine the relationship between frailty and vocal biomarkers in older people.

**Methods:**

Participants aged ≥ 60 years visiting geriatric outpatient clinics were recruited. They underwent frailty assessment (Cardiovascular Health Study [CHS] index; Study of Osteoporotic Fractures [SOF] index; and Fatigue, Resistance, Ambulation, Illness, and Loss of weight [FRAIL] index) and were asked to pronounce a sustained vowel /a/ for approximately 1 s. Four voice parameters were assessed: average number of zero crossings (A1), variations in local peaks and valleys (A2), variations in first and second formant frequencies (A3), and spectral energy ratio (A4).

**Results:**

Among 277 older adults, increased A1 was associated with a lower likelihood of frailty as defined by SOF (odds ratio [OR] 0.84, 95% confidence interval [CI] 0.74–0.96). Participants with larger A2 values were more likely to be frail, as defined by FRAIL and CHS (FRAIL: OR 1.41, 95% CI 1.12–1.79; CHS: OR 1.38, 95% CI 1.10–1.75). Sex differences were observed across the three frailty indices. In male participants, an increase in A3 by 10 points increased the odds of frailty by almost 7% (SOF: OR 1.07, 95% CI 1.02–1.12), 6% (FRAIL: OR 1.06, 95% CI 1.02–1.11), or 6% (CHS: OR 1.06, 95% CI 1.01–1.11). In female participants, an increase in A4 by 0.1 conferred a significant 2.8-fold (SOF: OR 2.81, 95% CI 1.71–4.62), 2.3-fold (FRAIL: OR 2.31, 95% CI 1.45–3.68), or 2.8-fold (CHS: OR 2.82, 95% CI 1.76–4.51, CHS) increased odds of frailty.

**Conclusions:**

Vocal biomarkers, especially spectral-domain voice parameters, might have potential for estimating frailty, as a non-invasive, instantaneous, objective, and cost-effective estimation tool, and demonstrating sex differences for individualised treatment of frailty.

**Supplementary Information:**

The online version contains supplementary material available at 10.1186/s12877-022-03237-7.

## Background

Frailty is a common issue in the aging population. Approximately one eighth to a quarter of older adults are estimated to be frail, whereas half are in the pre-frail stage [1]. Frailty is associated with adverse health outcomes and mortality [2, 3], placing a heavy burden on health- and aged-care systems [4].

Frailty is defined as ‘a medical syndrome with multiple causes and contributors that is characterised by diminished strength, endurance, and reduced physiologic function that increases an individual’s vulnerability for developing increased dependency and/or death’ [5]. Although frailty may be considered a geriatric syndrome of cumulative decline across multiple physiological systems, it does not yet have an internationally recognised standard definition [6]. For its practical application regardless of the theoretical definition used, frailty needs to be operationally defined. Researchers have measured the degree of frailty using a critical mass of phenotypic components in the hypothetical cycle of frailty (Cardiovascular Health Study [CHS] index) [7]. Furthermore, a simple frailty index with three components has been proposed based on the predictive validity of each component and its suitability for component assessment in clinical practice (Study of Osteoporotic Fractures [SOF] index) [8]. In addition, there is a frailty scale with four questions related to the components of the CHS index and one question (number of diseases) based on the Rockwood Clinical Frailty Scale [9] (Fatigue, Resistance, Ambulation, Illness and Loss of weight [FRAIL] index) [10]. These frailty measurements are widely recognised and commonly used in both clinical and population settings [6]. Although these frailty measurements have been well validated, generalisability issues remain [6]. Furthermore, there is a lack of consensus and international standard measurement for frailty, making the choice of the measurement tool difficult. Some frailty indices may be more applicable for use in population health studies, whereas others are best suited for clinical screening [11].

A good frailty measurement should fulfil several criteria. It should be able to accurately identify frailty based on the biological causative theory. Further, it should be able to provide consistent measurements without being influenced by outside factors. Frailty should also be measured in clinical practice as part of routine care to reduce health-care expenditures [12]. Finally, a frailty measurement tool should not be time consuming to use. On the basis of these criteria, vocal biomarkers might have potential for estimating frailty, as a non-invasive, instantaneous, objective, and cost-effective estimation tool [13].

The human voice reflects several diseases and pathological conditions owing to specific temporary or static changes that occur in the speech production organs or in the brain mechanisms controlling speech [13]. Thus, different acoustic parameters and patterns for diagnostic criteria can be derived from voice, mainly for detecting neurological and psychological conditions [14–16]. Recent studies have identified acoustic features for classifying symptoms according to body constitution [17], estimating lung obstruction [18], predicting adverse clinical outcomes among patients with congestive heart failure [19], and diagnosing diabetes mellitus [13]. However, the frailty syndrome is little discussed in the literature on the aging voice. Recent research has suggested “oral frailty,” a decline in masticatory and swallowing function associated with age-related changes [20] as well as deterioration in oral motor function (e.g., tongue pressure, oral diadochokinesis, and occlusal force) [21]. Oral frailty has been considered a possible independent frailty phenotype [21]. However, the association between oral frailty and voice changes remains unclear.

One study indicated that voice-related handicaps differ between robust and frail older adults, particularly on the exhaustion and weight loss domains of the Fried frailty criteria [22], whereas another study suggested a low correlation between voice dysfunction and frailty in nursing-home and assisted-living residents [23]. Given the mixed or scarce evidence, the current study aimed to determine the relationship between frailty and acoustic parameters among older adults.

## Methods

### Study design and sample

In this cross-sectional study, 277 participants aged ≥ 60 years who visited the geriatric outpatient clinic of a teaching hospital in middle Taiwan between January and December 2020 were recruited. Participants with acute infections and inflammatory diseases (eg, laryngopharyngitis, upper respiratory tract infection), anatomic lesions of the laryngopharynx, gastroesophageal reflux disease, neurologic diseases associated with voice disorders (eg, Parkinson disease, myasthenia gravis), or a surgical history involving the neck were excluded. The institutional review board approved the study protocol (CMUH108-REC3-160), and written informed consent was obtained from all participants.

### Frailty measurement

Three frailty scales were applied: CHS index (Fried’s frailty phenotype), SOF index, and FRAIL index. All characteristics of the original scales were retained in the present study. However, the measurements used to characterise the frailty criteria were slightly modified and operationalised as follows:

#### Fried’s frailty phenotype: CHS index [7]

- **Weight loss** was defined as unintentional weight loss of at least 4.5 kg or > 5% of the body weight in the previous year.
- **Fatigue/exhaustion** was measured using the question from the Center for Epidemiologic Studies Depression scale (‘In this last week, do you feel that you have less energy to do the things you want?’) and categorised as 0 (‘no’ answer) or 1 (‘yes’ answer).
- **Weakness** was assessed by measuring handgrip strength using cut-off values (for the dominant hand) modified for Asians (28 kg for men and 18 kg for women) [24].
- **Slowness** was evaluated using the walking time over a 4-m distance, with slow gait defined as a gait speed of < 1.0 m/s according to the 2019 Asian Working Group for Sarcopenia [25]. Participants who could not perform the walking test, such as wheelchair users, were classified as having low mobility.
- **Low physical activity** was assessed using the incidence and progression of basic activities of daily living disability from an emergency geriatric assessment [26], using the following question: ‘In last year, do you have any deterioration in activities of daily living (feeding, hygiene, dressing, transferring, walking, toileting, and bathing)?’. Participants who had difficulty performing at least one of the activities were considered not physically active. The association between physical activity and disability in activities of daily living has been confirmed in a previous study [27].

Participants were considered ‘frail’ if they fulfilled three or more criteria, ‘pre-frail’ if they fulfilled one or two criteria, and ‘robust’ if no criterion was fulfilled.

#### SOF index [8]

- **Weight loss** (intentional or unintentional weight loss > 5% of the body weight in the past year)
- **Chair stands** (inability to rise from a chair five consecutive times without using the arms)
- **Reduced energy level** (self-perceived reduced energy level as described by a negative answer to the question ‘do you feel full of energy?’ [28])

Participants were considered ‘frail’ if at least two of the three criteria were fulfilled, ‘pre-frail’ if only one criterion was present, and ‘robust’ if none of the criteria were present. The simple SOF index predicts the risk of falls, disability, hospitalisation, non-spine fractures, hip fractures, and death [8, 28–30]. Owing to its simplicity, the SOF index is widely used by the Taiwan Health Promotion Administration as a community screening tool [31].

#### FRAIL index [10]

The FRAIL index was developed by the International Association of Nutrition and Aging. It is a simple test that can identify frailty without face-to-face examination. All five components can be obtained from a comprehensive geriatric assessment [6, 10]. As four of the five components are from the CHS scale and one is from the Rockwood scale, we modified some assessment questions.

- **Fatigue** was measured by asking the respondents if they felt that they had less energy to do the things they wanted in the last week, with a ‘yes’ response scoring 1 point.
- **Resistance** was assessed by asking the participants if they had any difficulty rising from a chair five times without using the arms, with a ‘yes’ response scoring 1 point.
- **Ambulation** was assessed using gait speed, which has a prognostic capability comparable to that of walking distance for all-cause mortality in older patients with cardiovascular disease [32]. Moreover, it shows the highest effect size for discriminating between frailty subgroups, among other gait characteristics [33]. Ambulation was scored 1 in respondents with a gait speed of < 1.0 m/s.
- **Illness** was scored 1 in respondents who reported the use of eight drugs or more based on a core component in the emergency geriatric assessment [26].
- **Weight loss** was scored 1 in respondents with a self-reported weight reduction of ≥ 5% within the last 12 months.

The frailty scale scores range from 0 to 5 (ie, 1 point for each component; 0 = best to 5 = worst), with scores of 3–5, 1–2, and 0 indicating a frail, pre-frail, and robust health status, respectively [10].

### Acoustic parameters

The speech signals were digitised using a 16-bit A/D converter at a sampling rate of 10 kHz with an anti-aliasing function and analysed using LabVIEW. A sustained stable phonation of the vowel /a/ for approximately 1 s was chosen for the analysis. Four voice parameters, average number of zero crossings (A1), variations in local peaks and valleys (A2), variations in the first and second formant frequencies (A3), and spectral energy ratio (A4), were applied to analyse voice changes [34]. A1 was defined as the number of times the signal changed in value, from positive to negative, and vice versa, divided by the frame length. A2 was calculated as the average deviation of the largest (and the smallest) values for all peaks (and valleys), as a reflection of the degree of the temporal stability of vocal variations. A3 was defined as the average deviation from the mean of the first and second formant frequencies, which depend on the vocal tract length and the location and narrowness of constrictions along the vocal tract. A4 was defined as the ratio of the spectral energy above 3 kHz (end frequency) to the total spectral energy. A shift in spectral power to higher frequencies occurs in an indefinite formant structure [35].

### Statistical analysis

Statistical analyses were performed using the Stata software. Descriptive statistics were used to describe the study data, including absolute and percentage frequency distributions, mean and standard deviation. Univariate analysis was performed using one-way analysis of variance (ANOVA), with *p* < 0.05 indicating statistical significance. Each acoustic variable was separately evaluated in relation to the response of interest (frailty). Logistic regression was used for the elaboration of the prediction model. The odds ratios (ORs) and 95% confidence intervals (CIs) of the variables included in each model were calculated. The odds to predicted probabilities were also converted using the following formula: probability = odds/(1 + odds).

## Results

A total of 277 older adults were analysed. Frailty as defined by SOF, FRAIL, and CHS were all associated with older age, reduced body weight and body mass index, self-reported exhaustion, low muscle strength and resistance, slow gait speed, impairment in activities of daily living, polypharmacy, malnutrition, and emergency department visits or hospital admission (Table 1). Supplementary Table S1 shows the results of one-way ANOVA for the 15 factors considered, indicating significant differences (*p* < 0.05) for A1 (SOF), A2 (FRAIL), A2 (CHS), A3 (SOF), A3 (FRAIL), A3 (CHS), A4 (SOF), A4 (FRAIL), and A4 (CHS). These results suggest differences in the acoustic features between non-frail and frail older people.

**Table 1:** Characteristics of robust/prefrail and frail participants based on the Study of Osteoporotic Fractures (SOF) index, the Fatigue, Resistance, Ambulation, Illness and Loss of weight (FRAIL) index and the Cardiovascular Health Study (CHS) index

	SOF					FRAIL					CHS (Fried's)				
	Robust/ prefrail		Frail			Robust/ prefrail		Frail			Robust/ prefrail		Frail		
*N* = 277	*N*	(%)	*N*	(%)	*P* value†	*N*	(%)	*N*	(%)	*P* value†	*N*	(%)	*N*	(%)	*P* value†
Women (*N* = 175, 63.2%)	145	(63.6)	30	(61.2)	0.75	143	(64.1)	32	(59.3)	0.53	138	(62.7)	37	(64.9)	0.88
Age (years)^a^	73.8	± 6.7	76.6	± 8.3	0.01	73.8	± 6.8	76.4	± 8.0	0.02	73.6	± 6.7	76.9	± 8.1	< 0.01
Body weight (kg)^a^	59.6	± 10.5	50.7	± 8.4	< 0.01	59.1	± 10.3	53.5	± 11.1	< 0.01	59.2	± 9.6	53.6	± 13.1	< 0.01
BMI (kg/m^2^)^a^	24.4	± 4.2	21.6	± 3.0	< 0.01	24.2	± 4.1	22.6	± 4.1	0.01	24.1	± 3.8	23.0	± 5.1	0.07
Weight loss	24	(10.5)	34	(69.4)	< 0.01	26	(11.7)	32	(59.3)	< 0.01	33	(15.0)	25	(43.9)	< 0.01
Exhaustion	23	(10.1)	43	(87.8)	< 0.01	25	(11.2)	41	(75.9)	< 0.01	26	(11.8)	40	(70.2)	< 0.01
Low grip strength	102	(44.7)	30	(61.2)	< 0.01	95	(42.6)	37	(68.5)	< 0.01	81	(36.8)	51	(89.5)	< 0.01
Slow gait speed	64	(28.1)	35	(71.4)	< 0.01	56	(25.1)	43	(79.6)	< 0.01	46	(20.9)	53	(93.0)	< 0.01
Cannot complete 5 times CST	69	(30.3)	40	(81.6)	< 0.01	63	(28.3)	46	(85.2)	< 0.01	67	(30.5)	42	(73.7)	< 0.01
ADL impairment	16	(7.0)	14	(28.6)	< 0.01	10	(4.5)	20	(37.0)	< 0.01	3	(1.4)	27	(47.4)	< 0.01
Polypharmacy (> 8 kinds)	11	(4.8)	16	(32.7)	< 0.01	3	(1.4)	24	(44.4)	< 0.01	7	(3.2)	20	(35.1)	< 0.01
Malnutrition	77	(33.8)	29	(59.2)	< 0.01	71	(31.8)	35	(64.8)	< 0.01	71	(32.3)	35	(61.4)	< 0.01
ED visits or hospital admission in recent one year	12	(5.3)	12	(24.5)	< 0.01	10	(4.5)	14	(25.9)	< 0.01	5	(2.3)	19	(33.3)	< 0.01

^†^ Chi square/ Fisher’s test or ANOVA test. ^a^Mean ± standard deviation

*SOF* the Study of Osteoporotic Fractures index, *FRAIL* the Fatigue, Resistance, Ambulation, Illness and Loss of weight index, *CHS* the Cardiovascular Health Study index, *BMI* body mass index, *CST* chair stand test, *ADL* activity of daily life, *ED* emergency department

**Fig. 1:**
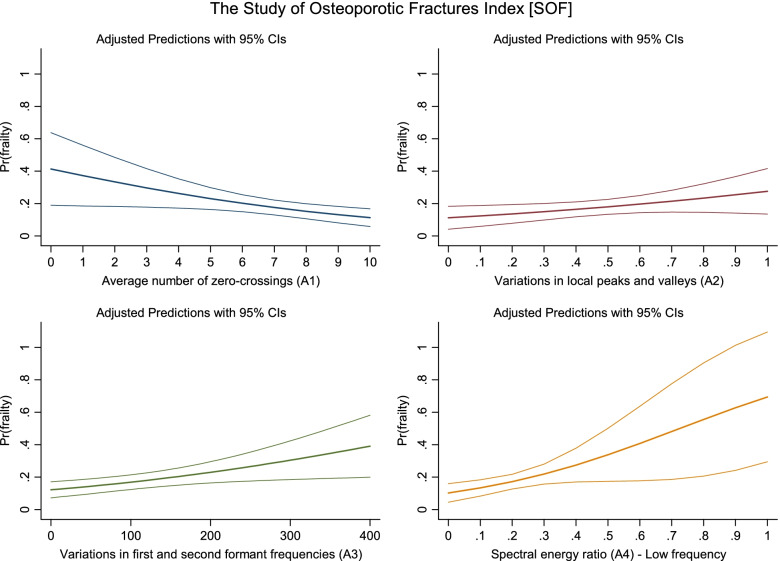
The association between acoustic features and the probability of frailty among older adults (the Study of Osteoporotic Fractures [SOF] Index), 2020. *Note*: upper left panel: odds ratio (OR) = 0.843^**^, 95% CI = 0.739–0.961; upper right panel: OR = 1.116^*^, 95% CI = 0.984–1.267; lower left panel: OR = 1.039^***^, 95% CI = 1.011–1.067; lower right panel: OR = 1.349^**^, 95% CI = 1.061–1.715. A1: OR for a one-unit change; A2: OR for a 0.1-unit change; A3: OR for a 10-unit change; A4: OR for a 0.1-unit change. All results were based on logistic regression analysis. ^*^ *p* < 0.10, ^**^ *p* < 0.05, and.^***^ *p* < 0.01

The acoustic features were related to the probability of frailty as defined by the SOF index among older adults. An increase in the A1 value was associated with a lower likelihood of frailty (OR 0.84, 95% CI 0.74–0.96) (Fig. 1), whereas an increase in A3 from 0 to 10 resulted in a 0.4 percentage point higher likelihood of frailty (OR 1.04, 95% CI 1.01–1.07). Respondents with larger A4 values had a higher likelihood of being frail (ie, a 3.1 percentage points higher frailty likelihood from 0 to 0.1) (OR 1.35, 95% CI 1.06–1.72). When the SOF criteria for frailty was applied, we found that the association between A2 and the frailty likelihood was not statistically significant.

**Fig. 2:**
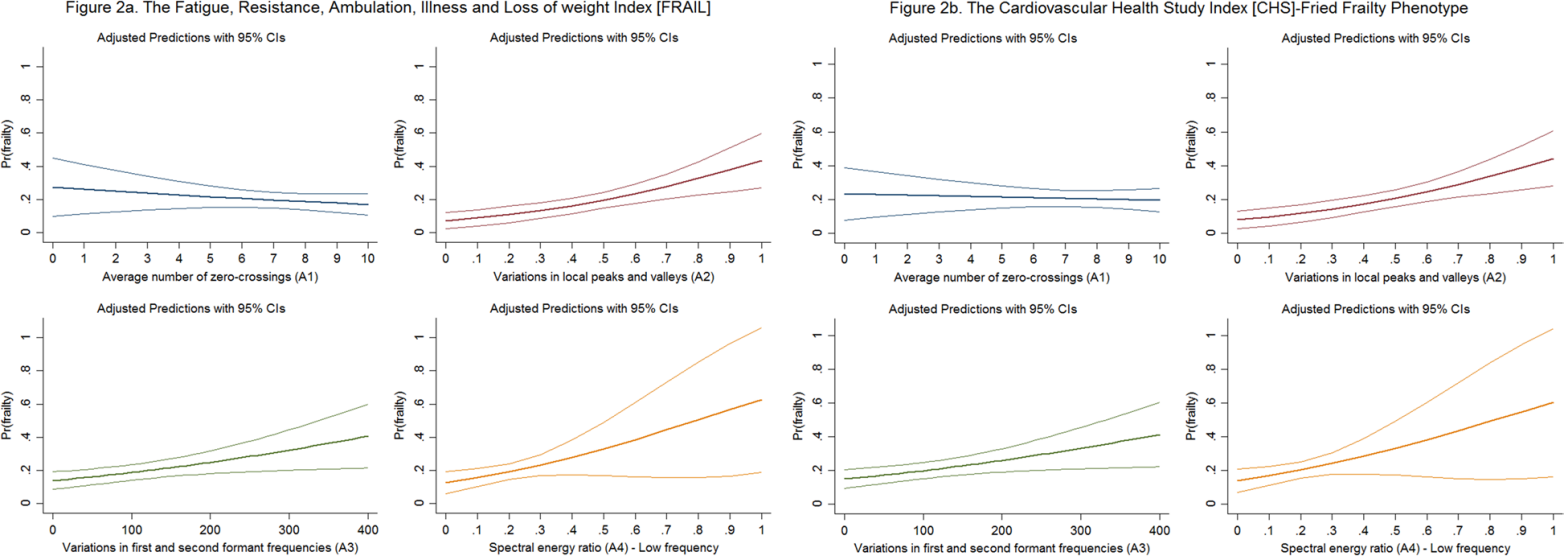
The association between acoustic features and the probability of frailty among older adults (the Fatigue, Resistance, Ambulation, Illness and Loss of weight [FRAIL] Index and Cardiovascular Health Study [CHS] Index), 2020. *Note*: Fig. 2a (the FRAIL Index): upper left panel: odds ratio (OR) = 0.941, 95% CI = 0.836–1.058; upper right panel: OR = 1.259^***^, 95% CI = 1.110–1.428; lower left panel: OR = 1.037^***^, 95% CI = 1.010–1.065; lower right panel: OR = 1.276^**^, 95% CI = 1.009–1.612; Fig. 2b (the CHS Index): upper left panel: odds ratio (OR) 0.978, 95% CI = 0.873–1.095; upper right panel: OR 1.248^***^, 95% CI = 1.103–1.413; lower left panel: OR 1.035^***^, 95% CI = 1.009–1.063; lower right panel: OR 1.249^*^, 95% CI = 0.992–1.573. A1: OR for a one-unit change; A2: OR for a 0.1-unit change; A3: OR for a 10-unit change; A4: OR for a 0.1-unit change. All results were based on logistic regression analysis. ^*^ *p* < 0.10, ^**^ *p* < 0.05, and.^***^ *p* < 0.01

Similar results were obtained when the frailty diagnosis was based on the FRAIL or CHS criteria. Respondents with larger A3 values were more likely to be frail (FRAIL: OR 1.04, 95% CI 1.01–1.06 (Fig. 2a); CHS: OR 1.04, 95% CI 1.01–1.06 (Fig. 2b)). Furthermore, the larger the A4 values of older adults, the higher the likelihood of frailty (FRAIL: OR 1.28, 95% CI 1.01–1.61 (Fig. 2a); CHS: OR 1.25, 95% CI 0.99–1.57, *p* = 0.059 (Fig. 2b)). Somewhat differently, an increase in A2 was associated with a higher likelihood of frailty (FRAIL: OR 1.26, 95% CI 1.11–1.43 (Fig. 2a); CHS: OR 1.25, 95% CI 1.10–1.41 (Fig. 2b)), whereas no statistically significant relationship was found between A1 and the frailty likelihood.

Supplementary Table S2 shows the sex-specific differences in the likelihood of frailty. When the SOF criteria were adopted, a stronger association between A1 and the frailty likelihood was observed in men (OR 0.73, 95% CI 0.56–0.94) than in women (OR 0.90, 95% CI 0.77–1.05). Meanwhile, on the basis of the FRAIL or CHS criteria for frailty, we found that men with larger A2 values were more likely to be frail (FRAIL: OR 1.41, 95% CI 1.12–1.79; CHS: OR 1.38, 95% CI 1.10–1.75); however, a weak positive relationship was found in women (FRAIL: OR 1.18, 95% CI 1.01–1.38; CHS: OR 1.19, 95% CI 1.03–1.39).

**Fig. 3:**
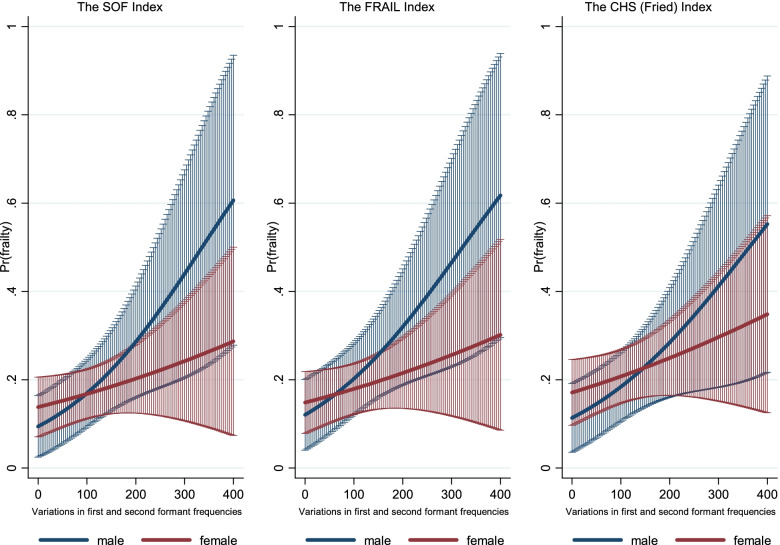
Gender difference in the association between variations in the first and second formant frequencies (A3) and the probability of frailty among older adults, by frailty index, 2020. *Note*: All results were based on logistic regression analysis. Results correspond to A3 of Supplementary Table S2, supplementary material. SOF: the Study of Osteoporotic Fractures index; FRAIL: the Fatigue, Resistance, Ambulation, Illness and Loss of weight index; CHS: the Cardiovascular Health Study index

**Fig. 4:**
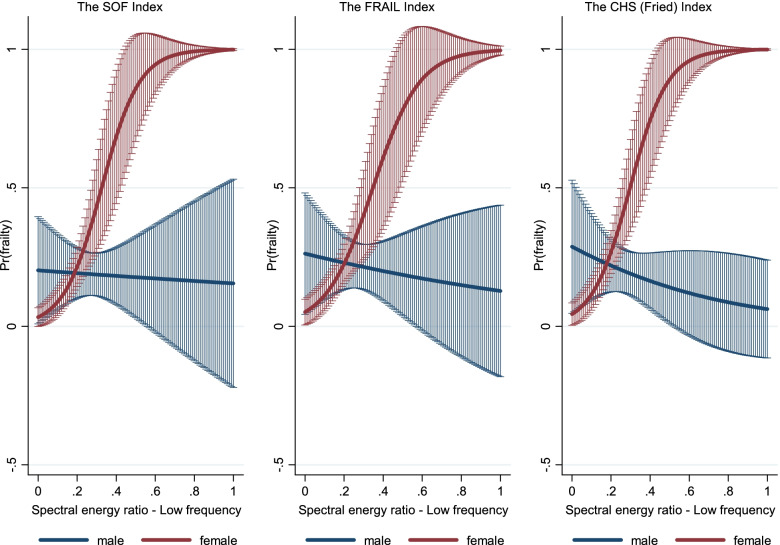
Gender difference in the association between spectral energy ratio (A4) and the probability of frailty among older adults, by frailty index, 2020. *Note*: All results were based on logistic regression analysis. Results correspond to A4 of Supplementary Table S2, supplementary material. SOF: the Study of Osteoporotic Fractures index; FRAIL: the Fatigue, Resistance, Ambulation, Illness and Loss of weight index; CHS: the Cardiovascular Health Study index

Similar sex differences were observed in older adults across the three frailty indices. In male older adults, an increase in A3 values by 10 points increased the odds of being frail by almost 7% (SOF: OR 1.07, 95% CI 1.02–1.12), 6% (FRAIL: OR 1.06, 95% CI 1.02–1.11), or 6% (CHS: OR 1.06, 95% CI 1.01–1.11) (Fig. 3). In female older adults, an increase in A3 was associated with a slightly higher likelihood of frailty, which was not significant (*p* > 0.05). In female older adults, an increase in A4 by 0.1 conferred a significant 2.8-fold (SOF: OR 2.81, 95% CI 1.71–4.62), 2.3-fold (FRAIL: OR 2.31, 95% CI 1.45–3.68), or 2.8-fold (CHS: OR 2.82, 95% CI 1.76–4.51) increased odds of being frail (Fig. 4). However, an opposite effect was observed in male older adults, but without statistical significance.

## Discussion

In this study, we determined, for the first time to our knowledge, the relationship between frailty syndrome and acoustic measures in older adults. We found that the acoustic features differed according to the frailty status. Of the four evaluated acoustic measures, A1 was found to be more related to frailty as defined by the SOF index, whereas A2 was more related to frailty as defined by the FRAIL and CHS indices. Moreover, we found sex differences in the A3 and A4 values.

The currently accepted mechanism of phonation is that the interaction of aerodynamic forces and the mechanical properties of laryngeal tissues generate vocal sounds [36]. The reason for the temporal parameters A1 (average number of zero crossings) and A2 (variations in local peaks and valleys) being more strongly associated with frailty defined by SOF and CHS/FRAIL, respectively, might be related to each component of these frailty indices. First, the determining component in the SOF index (reduced energy level) is associated with a feeling of constant tiredness or weakness that leads to a decrease in the aerodynamic forces required to produce phonation. In this way, participants who were more prone to fatigue performed fewer average numbers of zero crossings (A1) (Fig. 1). Second, muscle mass and strength, which account for a large proportion of both the CHS and FRAIL indices, are associated with aerodynamic stability. Older adults with frailty as defined by the CHS or FRAIL index had larger variations in local peaks and valleys (A2) (Fig. 2), corresponding to impaired aerodynamic force control [37]. Owing to muscular and phonatory compensatory mechanisms in older adults, greater expansion of the chest and lungs and more abdominal movement are required to increase vocal amplitude [38, 39]. Moreover, phonation is initiated at a higher lung volume [40]. These situations result in larger variations in the local peaks and valleys. Therefore, aerodynamic stability, rather than the strength of aerodynamic forces, is more relevant to muscle dysfunction in relation to frailty in older adults. The potential mechanisms, however, need to be backed by further evidence.

A3 and A4, the frequency-domain voice parameters generated from Fourier analysis, can better identify frailty in older adults than the time-domain voice parameters (A1 and A2). Frequency-domain voice parameters account for interactions between the vocal folds and the glottal system [41]. For example, the frail group presented significantly higher A4 values (Figs. 1 and 2). This may be because loss of mass and strength in muscles that control the vocal cords contributes to a longer open phase of the glottis, which subsequently leads to a more dominant first harmonic in the low-frequency portion of the voice source spectrum, thus increasing the energy in the low-frequency portion of the source spectrum [41]. Another possible mechanism is that reduced glottal flow causes an increase in the time for opening the glottis, thereby increasing the low-frequency energy [42]. Loss of muscle strength and mass and reduced glottal flow are the main features of the three frailty indices. Thus, A4 may be a good acoustic parameter for assessing frailty.

More specifically, we found sex differences in the spectral characteristics of phonation (A3 and A4) but not in the temporal parameters (A1 and A2) across the three frailty indices (supplementary Table S2). The variations in the first and second formant frequencies (A3) are closely tied to the interplay of glottal airflow and vocal fold vibration (controlled by tiny muscles in the larynx called the thyroarytenoid and cricothyroid muscles) that can generate vocal tract resonances [43]. Among older men, the frail group presented significantly higher A3 values (Fig. 3), reflecting resonance frequency instability for the first and second formants, which can be attributed to insufficient airflow or poor vocal cord control via the laryngeal muscles. In contrast, a relatively small increase in A3 was found in frail older women, possibly because of lower muscle strength, reduced ability to control muscle forces, and lower glottal airflow in robust older women than in their male counterparts. Furthermore, the spectral energy ratio at low frequencies (A4), which is influenced by the vocal tract structure [41], was found to be a more sensitive parameter in the diagnosis of frailty in older women (Fig. 4). Frailty could be characterised by a lack of tension within the vocal cords due to atrophic changes in the thyroarytenoid muscle, a paired skeletal muscle that makes up the bulk of the true vocal fold body and manages tension along the vocal fold edge [44]. As a result, aerodynamic forces could not sufficiently generate vocal cord vibrations, which, in turn, will result in a longer open phase of the glottis and an energy increase in the low-frequency portion [42]. However, there is a sex difference in spectral tilt, in that robust men tend to have more spectral energy in the low-frequency portion of the source spectrum than their female counterparts. Thus, changes in A4 values are more prevalent in frail women. However, this remains mainly speculative, and we require further evidence for the claim.

This study had several limitations. First, although previous studies have shown that the choice of the sustained vowel /a/ as an acoustic measure has some advantages (eg, it can be pronounced by any person without training and is relatively stable for analysis [34]), some acoustic features are not captured in a 1-s duration. Second, steady vowel utterances bear limited resemblance to natural language production, which requires dynamic adjustments to voice frequency and amplitude. Third, recent research has investigated the cross-sectional area of the geniohyoid muscle, tongue pressure, and oral diadochokinesis as an index of oral sarcopenia [45]. In future studies, we can test the mechanism of anatomical changes that occur in frailty that lead to alterations in the acoustic properties of the voice. Fourth, because of the cross-sectional design, we cannot establish a causal relationship, and the context of a single centre may limit the generalisability of the results. Finally, although the three frailty tools successfully recognised the physical dimension of frailty, they failed to consider the impact of psychological and social function in the development and progression of frailty and its impact on outcomes such as acoustic features. Further studies need to apply an integral definition of frailty consisting of physical, psychological, and social components (eg, Tilburg Frailty Indicator) and examine its relationship with voice-related measures.

At least two implications warrant consideration. First, efforts to link acoustic measures to the diagnosis of frailty in older adults could start with the two vital acoustic parameters, A3 and A4, which considerably better match the natural voice than A1 and A2. Second, assessing frailty through A3 and A4 might be more effective if the use of such strategies is conditional on sex differences. In summary, frailty is a complex phenotype seen with aging that is associated with a feeling of fatigue and loss of muscle mass and function. Therefore, spectral-domain voice parameters are likely useful tools that are worthy of attention.

## Conclusions

Given that frailty syndrome is little discussed in the literature on the aging voice, the current study, to the best of our knowledge, the first to determine the relationship between frailty and acoustic parameters among older adults. They might be useful in frailty diagnosis in older adults. Vocal biomarkers, especially spectral-domain voice parameters, might have potential for estimating frailty, as a non-invasive, instantaneous, objective, and cost-effective estimation tool, and demonstrating sex differences for individualised treatment of frailty.

## Supplementary Information

**Additional file 1:** **Supplementary Table S1.** A one-way analysis of variance on acoustic features differences, 2020. **Supplementary Table S2.** Sex-specific differences in the association between acoustic features and the probability of frailty among older adults, by the three frailty indices, 2020.

## Data Availability

The datasets used and/or analysed during the current study are available from the corresponding author on reasonable request.

## Abbreviations

A1: Average number of zero crossings
A2: Variations in local peaks and valleys
A3: Variations in first and second formant frequencies
A4: Spectral energy ratio
ANOVA: Analysis of variance
CHS: Cardiovascular Health Study
CI: Confidence interval
FRAIL: Fatigue, Resistance, Ambulation, Illness, and Loss of weight
OR: Odds ratio
SOF: Study of Osteoporotic Fractures
