# Supplementary material for: Predicting frailty in older adults using vocal biomarkers: a cross-sectional study
Source: BMC Geriatr. 2022 Jul 1;22:549. doi: 10.1186/s12877-022-03237-7 (PMC9248103; doi:10.1186/s12877-022-03237-7)
Supplement: Supplementary file 1 — Additional file 1: Supplementary Table S1. A one-way analysis of variance on acoustic features differences, 2020. Supplementary Table S2. Sex-specific differences in the association between acoustic features and the probability of frailty among older adults, by the three frailty indices, 2020. [file 12877_2022_3237_MOESM1_ESM.docx]

**Supplementary Table S1**. A one-way analysis of variance on acoustic features differences, 2020

|  | Mean | F | p value |
| --- | --- | --- | --- |
| A1 (SOF) | 0: 7.495 | 6.75 | 0.010 |
|  | 1: 6.439 |  |  |
| A1 (FRAIL) | 0: 7.387 | 1.04 | 0.308 |
|  | 1: 6.983 |  |  |
| A1 (CHS) | 0: 7.339 | 0.15 | 0.696 |
|  | 1: 7.187 |  |  |
| A2 (SOF) | 0: 0.450 | 2.97 | 0.086 |
|  | 1: 0.514 |  |  |
| A2 (FRAIL) | 0: 0.436 | 14.49 | 0.000 |
|  | 1: 0.569 |  |  |
| A2 (CHS) | 0: 0.435 | 13.88 | 0.000 |
|  | 1: 0.563 |  |  |
| A3 (SOF) | 0: 94.483 | 8.49 | 0.004 |
|  | 1: 139.790 |  |  |
| A3 (FRAIL) | 0: 94.321 | 7.83 | 0.006 |
|  | 1: 136.264 |  |  |
| A3 (CHS) | 0: 94.317 | 7.31 | 0.007 |
|  | 1: 134.071 |  |  |
| A4 (SOF) | 0: 0.190 | 6.30 | 0.013 |
|  | 1: 0.237 |  |  |
| A4 (FRAIL) | 0: 0.191 | 4.29 | 0.039 |
|  | 1: 0.228 |  |  |
| A4 (CHS) | 0: 0.191 | 3.67 | 0.050 |
|  | 1: 0.225 |  |  |

*Note*: 0: robust or prefrail; 1: frail. A1: average number of zero-crossings; A2: variations in local peaks and valleys; A3: variations in the first and second formant frequencies; A4: spectral energy ratio - low frequency.

**Supplementary Table S2**. Sex-specific differences in the association between acoustic features and the probability of frailty among older adults, by the three frailty indices, 2020

|  | SOF | | FRAIL | | CHS (Fried) | |
| --- | --- | --- | --- | --- | --- | --- |
|  | Male | Female | Male | Female | Male | Female |
|  | OR | OR | OR | OR | OR | OR |
|  | (95% CI) | (95% CI) | (95% CI) | (95% CI) | (95% CI) | (95% CI) |
| A1 | 0.728^*^ | 0.901 | 0.839 | 0.999 | 0.960 | 0.987 |
|  | (0.565-0.937) | (0.772-1.053) | (0.677-1.039) | (0.866-1.153) | (0.785-1.175) | (0.861-1.131) |
| A2 | 1.153 | 1.093 | 1.414^*^ | 1.184^*^ | 1.384^*^ | 1.195^*^ |
|  | (0.931-1.428) | (0.933-1.280) | (1.117-1.788) | (1.014-1.382) | (1.096-1.747) | (1.030-1.386) |
| A3 | 1.070^*^ | 1.023 | 1.064^*^ | 1.023 | 1.058^*^ | 1.024 |
|  | (1.020-1.121) | (0.989-1.059) | (1.016-1.114) | (0.989-1.058) | (1.011-1.108) | (0.992-1.058) |
| A4 | 0.968 | 2.814^*^ | 0.915 | 2.309^*^ | 0.835 | 2.816^*^ |
|  | (0.655-1.432) | (1.713-4.622) | (0.628-1.334) | (1.447-3.685) | (0.557-1.251) | (1.759-4.507) |

*Note*: **p* < .05. OR: odds ratio; A1: average number of zero-crossings; A2: variations in local peaks and valleys; A3: variations in the first and second formant frequencies; A4: spectral energy ratio - low frequency. A1: OR for a one-unit change; A2: OR for a 0.1-unit change; A3: OR for a 10-unit change; A4: OR for a 0.1-unit change. SOF: the Study of Osteoporotic Fractures index; FRAIL: the Fatigue, Resistance, Ambulation, Illness and Loss of weight index; CHS: the Cardiovascular Health Study index.
